# Androgen Receptor-Related Non-coding RNAs in Prostate Cancer

**DOI:** 10.3389/fcell.2021.660853

**Published:** 2021-04-01

**Authors:** Yongyong Yang, Kilia Y. Liu, Qi Liu, Qi Cao

**Affiliations:** ^1^Department of Urology, Feinberg School of Medicine, Northwestern University, Chicago, IL, United States; ^2^Robert H. Lurie Comprehensive Cancer Center, Feinberg School of Medicine, Northwestern University, Chicago, IL, United States

**Keywords:** prostate cancer, androgen receptor, non-coding RNA, microRNA, lncRNA, circRNA

## Abstract

Prostate cancer (PCa) is the second leading cause of cancer-related death among men in the United States. Androgen receptor (AR) signaling is the dominant oncogenic pathway in PCa and the main strategy of PCa treatment is to control the AR activity. A large number of patients acquire resistance to Androgen deprivation therapy (ADT) due to AR aberrant activation, resulting in castration-resistant prostate cancer (CRPC). Understanding the molecular mechanisms underlying AR signaling in the PCa is critical to identify new therapeutic targets for PCa patients. The recent advances in high-throughput RNA sequencing (RNA-seq) techniques identified an increasing number of non-coding RNAs (ncRNAs) that play critical roles through various mechanisms in different diseases. Some ncRNAs have shown great potentials as biomarkers and therapeutic targets. Many ncRNAs have been investigated to regulate PCa through direct association with AR. In this review, we aim to comprehensively summarize recent findings of the functional roles and molecular mechanisms of AR-related ncRNAs as AR regulators or targets in the progression of PCa.

## Introduction

Prostate cancer (PCa) is the most frequently diagnosed cancer and the second-highest cause of cancer death among men in the United States, with an estimated 248,530 new cases and 34,130 deaths expected in 2021 in the United States (Siegel et al., 2021). The growth and survival of PCa are mainly dependent on the sex steroid hormone, androgens (Folkerd and Dowsett, 2010). The androgen receptor (AR) is a ligand-activated transcription factor that is vital for both normal prostate development and tumorigenesis. Upon binding by androgen in the cytoplasm, AR dimerizes and translocates to the nucleus, stimulating target gene transcription through association with androgen response elements (AREs) within promoter and enhancer sequences. AR and its downstream signal cascades are critical for the initiation and progression of both localized and advanced metastatic PCa (Scher and Sawyers, 2005). Advances in screening and therapeutic strategies promoted successful treatment of PCa by surgery and/or radiation. The testing of prostate-specific antigen (PSA), a prototypic AR target, has been used for years as a diagnostic biomarker for the disease (Lilja et al., 2008). Androgen deprivation therapy (ADT) by AR antagonists and chemical castration is the standard treatment for patients with biochemical recurrence after primary therapy or with locally advanced or metastatic disease (Feldman and Feldman, 2001). Patients with metastasis-free PCa have a 100% 5-year survival rate (Brawley, 2012). Unfortunately, the majority of primary cancers will eventually acquire ADT resistance and progress to castration-resistant prostate cancer (CRPC) (Kirby et al., 2011). Patients with metastatic PCa have a low 5-year survival rate (Kirby et al., 2011; Brawley, 2012). Generally, CRPC is caused by AR aberrant activation with enhanced AR expression, hypersensitivity to androgens (Waltering et al., 2009), intra-tumoral steroidogenesis (Locke et al., 2008), and abnormal AR splicing variant expression (Sun et al., 2010). Recent studies revealed that the frequency of AR-negative neuroendocrine prostate cancer (NEPC) and AR-Null and Neuroendocrine-Null Prostate Cancer (Double-Negative PCa, DNPC) is elevated due to the application of potent AR antagonists such as enzalutamide (ENZ) and abiraterone (Aparicio et al., 2011; Hu et al., 2015; Bluemn et al., 2017; Labrecque et al., 2019). Therefore, there is an urgent need to elucidate the molecular mechanisms contributing to the development of AR-dependent CRPC as well as advanced NEPC and DNPC for developing alternative therapeutic options for advanced PCa.

Next-generation RNA sequencing (RNA-seq) advances have become overly convenient to measure gene expression levels and explore new transcriptional units across the transcriptome. 2% of the human genome encodes approximately 20,000 protein-coding genes (Lander et al., 2001; Venter et al., 2001; Alexander et al., 2010; Consortium, 2012) and up to 80% of the human genome encodes a large number of non-coding RNAs (ncRNA) (Consortium, 2012; Djebali et al., 2012). Ribosomal RNA (rRNA) (about 80% of the total RNA weight) and transfer RNA (tRNA) (about 15% of the total RNA weight) are two of the most abundant ncRNA types in cells (Palazzo and Lee, 2015). The other ncRNAs are categorized as short ncRNAs (sncRNAs) and long ncRNAs (lncRNAs) based on whether their size is longer than 200 bases (Cech and Steitz, 2014). SncRNAs include microRNA (miRNA) (Lagos-Quintana et al., 2001), small interfering RNA (siRNA) (Hamilton and Baulcombe, 1999), piwi-interacting RNA (piRNA) (Siomi et al., 2011), small nucleolar RNA (snoRNA) (Kiss, 2001; Matera et al., 2007), small nuclear RNA (snRNA) (Matera et al., 2007; Guiro and Murphy, 2017), and tRNA-derived fragments (tRF) (Schimmel, 2018). Unlike linear lncRNAs, circular RNAs (circRNAs) are single-stranded circularized ncRNAs commonly generated from the precursor mRNA (pre-mRNA) back-splicing process (Memczak et al., 2013; Salzman, 2016). Functionally, rRNA, tRNA, snoRNA, and snRNA are housekeeping ncRNAs, while miRNA, siRNA, piRNA, tRF, lncRNA, and circRNA are regulatory transcripts.

Over recent decades, ncRNAs have been emerged as critical regulators instead of junk RNAs in different disease processes, including cancer (Du et al., 2013; Hayes et al., 2014; Vo et al., 2019). Notably, many ncRNAs (miRNA, lncRNA, and circRNA) are aberrantly expressed with significant contribution to PCa initiation and/or progression (Bonci et al., 2008; Prensner et al., 2011; Pickl et al., 2014; Fredsoe et al., 2018; Hua et al., 2018; Chen S. et al., 2019). MiR-101 negatively regulates *EZH2* expression by binding to *EZH2* 3′ untranslated region (UTR), and has a strong negative correlation with PCa progression from benign to localized disease to metastasis (Varambally et al., 2008). LncRNA *SChLAP1* is critical for PCa cell invasiveness and metastasis through antagonizing the genome-wide localization and regulatory functions of the SWI/SNF chromatin-modifying complex (Prensner et al., 2013). CircRNA 0005276 (circ0005276), a circular RNA stem from *XIAP*, is highly expressed in PCa tissues with advanced tumor stage and metastasis. Circ0005276 interacts with FUS to regulate the transcription of *XIAP* in PCa, thus promoting the tumorigenesis and development of PCa (Feng et al., 2019). Aside from miRNAs, lncRNAs and circRNAs are relatively new players in the ncRNA field and are less well understood. ncRNAs are gaining widespread attention for their abundance in number, expression specificity, functional roles in diseases, and potential clinical applications. Given the critical role of AR in PCa initiation and progression, we will focus on the impact of miRNA, lncRNA, and circRNA, on AR’s function in PCa in this review.

## ncRNAs as AR Regulators

### AR Is Repressed by miRNAs

MicroRNAs are a family of small untranslated RNAs with ∼21–25 nucleotides in size that control gene expression by mediating target mRNA degradation (He and Hannon, 2004; Bartel, 2009), and repressing (Pillai et al., 2005; Petersen et al., 2006; Mathonnet et al., 2007) or promoting (Vasudevan et al., 2007; Truesdell et al., 2012) target mRNA translation. These regulations often occur through the association of miRNAs with 3′ UTRs of transcripts (Bartel, 2009; Krol et al., 2010), and some miRNAs may also target 5′ UTR and coding regions of transcripts. Many tools have been developed to predict miRNA targets (Chen L. et al., 2019). *AR* mRNA is comprised of a 1.1 kb 5′ UTR, a 2.7 kb open reading frame (ORF), and an exceptionally long 3′ UTR with a length of approximately 6.8 kb (Ostling et al., 2011; Ebron and Shukla, 2016). Thus *AR* mRNA is the most miRNA targeted transcript in PCa cells (Hamilton et al., 2016; Table 1).

**TABLE 1:** MiRNAs targeting *AR* mRNA.

MiRNAs (References)	Expression in PCa	Target region of *AR* mRNA	Cancer types	Function in cancer cells
miR-124 (Shi et al., 2013, 2015; Xiong et al., 2017)	↓	3′UTR	PCa, bladder cancer	Inhibits cell growth and increases apoptosis
miR-135b (Ostling et al., 2011; Aakula et al., 2015; Bao S. X. et al., 2020)	↓	3′UTR	PCa, hepatocellular carcinoma (HCC), breast cancer	Inhibits cell growth
miR-181c-5p (Wu et al., 2019)	–	3′UTR	PCa	Increases ENZ sensitivity of PCa cells and represses cell invasion
miR-185 (Ostling et al., 2011; Qu et al., 2013; Liu C. et al., 2015; Kalinina et al., 2020)	↓	3′UTR	PCa, breast cancer	Suppresses cell growth, migration, invasion, and tumorigenicity
miR-193a-3p (Kumar et al., 2016; Liu et al., 2017)	↓	Coding region	PCa	Suppresses cell growth
miR-197-3p (Fletcher et al., 2019; Huang et al., 2020)	↓	3′UTR	PCa	Inhibits cell growth and colony formation
miR-205 (Boll et al., 2013; Hagman et al., 2013; Coarfa et al., 2016; Kalinina et al., 2020)	↓	3′UTR	PCa, breast cancer	Suppresses cell growth, migration, and invasion
miR-297 (Ostling et al., 2011; Fang et al., 2016)	↓	3′UTR	PCa	Suppresses cell growth
miR-299-3p (Ostling et al., 2011; Ganapathy et al., 2020)	↓	3′UTR	PCa	Suppresses cell growth, migration, induces cell cycle arrest, and apoptosis
miR-30b-3p (Kao et al., 2014; Kumar et al., 2016)	↓	3′UTR	PCa	Suppresses EMT phenotypes and inhibits cell migration and invasion
miR-31 (Lin et al., 2013; Coarfa et al., 2016; Wang et al., 2016)	↓	Coding region	PCa	Suppresses cell growth, migration and invasiveness
miR-320a (Okato et al., 2016; Sato et al., 2016; Lieb et al., 2018)	↓	3′UTR	PCa	Suppresses cell growth, migration and invasiveness
miR-320b (Hsieh et al., 2013; Lieb et al., 2018; Dai et al., 2019)	↓	3′UTR	PCa	Suppresses cell growth
miR-346 (Fletcher et al., 2019; Lin et al., 2020)	↑	3′UTR	PCa	Promotes cell growth and invasion
miR-34a (Liu et al., 2011; Ostling et al., 2011; Kashat et al., 2012; Leite et al., 2015)	↓	3′UTR	PCa	Inhibits prostate cancer stem cell regeneration and metastasis
miR-34c (Hagman et al., 2010; Ostling et al., 2011; Walter et al., 2013; Fang et al., 2016)	↓	3′UTR	PCa	Suppresses cell growth
miR-361-3p (Fletcher et al., 2019; Liu B. et al., 2020)	↓	3′UTR	PCa	Suppresses cell growth and increases ENZ sensitivity of PCa cells
miR-371-3p (Ostling et al., 2011; Leite et al., 2015; Kumar et al., 2016)	↓	Coding region, 3′UTR	PCa	Suppresses cell growth
miR-421 (Ostling et al., 2011; Meng et al., 2016)	↓	3′UTR	PCa	Suppresses cell growth, induces cell cycle arrest, reduces glycolysis, and inhibits migration
miR-449a (Ostling et al., 2011; Zheng et al., 2015; Chen W. et al., 2017; Guo et al., 2018)	↓	3′UTR	PCa, bladder cancer	Suppresses cell growth, invasion, and angiogenesis
miR-449b (Ostling et al., 2011; Mortensen et al., 2014)	↑	3′UTR	PCa	Suppresses cell growth
miR-488^∗^ (Sikand et al., 2011; Ebron and Shukla, 2016)	-	3′UTR	PCa	Suppresses cell growth, increases apoptosis
miR-541-3p (Kumar et al., 2016; He et al., 2021)	↓	3′UTR	PCa	Suppresses cell growth and enhances the radiosensitivity of PCa cells
miR-634 (Ostling et al., 2011; Kumar et al., 2016)	–	3′UTR	PCa	Suppresses cell growth
miR-646 (Kumar et al., 2016)	–	Coding region 3′UTR	PCa	Suppresses cell growth
miR-654-5p (Ostling et al., 2011; Kumar et al., 2016)	–	3′UTR	PCa	Suppresses cell growth
miR-92a-2-5p (Liu G. et al., 2020)	–	3′UTR	HCC	Increases liver cancer cell invasion
miR-9-5p (Ostling et al., 2011; Kumar et al., 2016; Moazzeni et al., 2017; Bandini et al., 2020)	–	3′UTR	PCa, breast cancer	Suppresses cell growth

Several groups have systemically explored AR modulatory miRNAs through different strategies (Ostling et al., 2011; Hamilton et al., 2016; Kumar et al., 2016; Fletcher et al., 2019). Ostling et al. (2011) and Kumar et al. (2016) used pre-miR libraries to perform gain-of-function screening for AR-modulatory miRs in PCa cells. Östling et al. identified and validated 13 miRNAs that interact with the *AR* 3′ UTR region and could significantly reduce *AR* 3′ UTR activity: miR-135b, miR-185, miR-297, miR-299-3p, miR-34a, miR-34c, miR-371-3p, miR-421, miR-449a, miR-449b, miR-634, miR-654-5p, and miR-9. Among these miRNAs, miR-185 and miR-34a were consistently reported by other groups to show the regulatory function on PCa aggressiveness through directly targeting *AR* (Kashat et al., 2012; Qu et al., 2013). Similarly, Kumar et al. (2016) identified 15 miRNAs (miR-101-3p, miR-138-5p, miR-149-3p, miR-30b-3p, miR-30c-5p, miR-30d-5p, miR-411-3p, miR-425-5p, miR-488-5p, miR-541-3p, miR-635, miR-646, miR-650, miR-654, and miR-9-5p) that significantly suppressed *AR* 3′ UTR reporter activity, especially miR-9-5p, miR-30b-3p and miR-541-3p, highlighting the critical role of miR-30 family members in inhibiting AR activity through 3′ UTR association. The study also revealed that 3 miRNAs, miR-371-3p, miR-193-3p, and miR-646 could suppress *AR* transcriptional activity through binding sites within the coding region of *AR* mRNA (Kumar et al., 2016). The role of miR-488 in inhibiting AR expression in PCa cells was also confirmed by Sikand et al. (2011). Hamilton et al. (2016) applied photoactivatable ribonucleoside-enhanced cross-linking immunoprecipitation of the Argonaute protein and sequencing (AGO-CLIP-Seq) to broadly explore interactions between miRNAs and miRNA target sites in a panel of PCa cells (Hamilton et al., 2016). Among 22 PCa driver genes, *AR* 3′ UTR has the most abundant miRNA target sites (71 unique miRNA families at 147 seed sites), including the miR-135, miR-185, miR-34, miR-421, and miR-9 families reported by Ostling et al. (2011).

Recently, using a library of LNA-modified antisense inhibitors against 983 human miRNAs, Fletcher et al. (2019) systematically identified microRNAs modulating AR activity in PCa cells. The application of the miR inhibitors limits off-target or non-specific effects by avoiding targeting endogenous miRNA processing or effector complexes. 78 miRNA inhibitors were found to significantly modulate AR reporter activity, including inhibitors of miR-135b, miR-421, miR-449a, miR-634, and miR-654-5p, which is consistent with Ostling et al.’s (2011) report. Interestingly, inhibition of miR-346, miR-361-3p, and miR-197 significantly reduced AR activity in a dose-dependent manner. Upregulation of *AR* 3′ UTR activity by miR-346, miR-361-3p, and miR-197 was also confirmed by *AR* 3′ UTR reporter assay combined with miRNA mimics. Mimics of miR-346, miR-361-3p, and miR-197 also prevented Actinomycin D-induced loss of *AR* transcript. Previous studies demonstrated that miR-346 binds to 3′ UTR of *AGO2* and *hTERT*. Upon miR-346 binding to the 3′ UTR of *AGO2* and *hTERT*, the middle sequence motif (CCGCAU) of miR-346 recruits G-rich RNA sequence binding factor 1 (GRSF1) to form a “bulge loop,” thus facilitating the recruitment of *AGO2* mRNA and *hTERT* mRNA to ribosomes to promote translation (Guo et al., 2015; Song et al., 2015). MiR-346, miR-361-3p, and miR-197 may enhance *AR* mRNA stability and promote AR expression through a similar mechanism. In Fletcher et al.’s (2019) study, miR-197 inhibitor increased caspase activity and suppressed cell growth, indicating miR-197 may act as a tumor promoter. However, miR-197’s expression was lower in prostate tumor tissues than in normal tissues (Fletcher et al., 2019), which is consistent with the miR-197 inhibitor’s role in promoting PCa cell growth reported by Huang et al. (2020). Besides, the miR-361-3p inhibitor also suppressed PCa cell growth (Fletcher et al., 2019), while another report showed that miR-361-3p enhanced ENZ sensitivity of PCa cells and inhibited PCa cell growth by suppressing AR splice variant 7 (*AR-V7*) expression (Liu B. et al., 2020). Thus, the function of miR-197 and miR-361-3p in PCa cell growth from different studies are conflicted. Some miRNAs may be able to either activate or repress mRNA translation, and whether they act as translation activators or repressors highly depends on cell cycle and RNA binding factors (Vasudevan et al., 2007; Truesdell et al., 2012). Further studies are necessary to disentangle these contradictory results.

Many miRNAs targeting *AR* mRNA might be not reflected in the result from the above systematic analysis (Table 1). MiR-205 is mainly expressed in prostate basal epithelial cells (Zhang et al., 2010; Gandellini et al., 2012), and PCa patients with low expression of miR-205 have poor survival (Hagman et al., 2013). MiR-205 can suppress AR expression by binding to *AR* 3′ UTR, and also interfere with MAPK and IL-6 signaling pathways in PCa cells (Boll et al., 2013; Hagman et al., 2013), while the promoter region of the miR-205 gene contains ARE and the expression of miR-205 is increased after AR activation by R1881 treatment (Hagman et al., 2013). MiR-212 is downregulated in prostate tumor cells, and it suppresses the transcription of *AR* and *AR-V7* through direct targeting *hnRNPH1*, which may regulate *AR* mRNA transcription or splicing. Interestingly, hnRNPH1 can also interact with AR protein and modulate AR binding to target genes (Yang et al., 2016). MiR-31 is regulated by promoter hypermethylation in both triple-negative breast cancer (Augoff et al., 2012) and PCa (Lin et al., 2013), and its expression is negatively correlated with the aggressiveness of the PCa. MiR-31 directly inhibits AR expression through binding to the coding region of *AR* mRNA. Subsequently, genes related to cell cycle regulation are also repressed by miR-31 as its direct targets. Interestingly, miR-31 can be suppressed by AR as a transcriptional target, forming a negative regulation loop between miR-31 and AR (Lin et al., 2013). MiR-124 was downregulated in PCa (Shi et al., 2013, 2015), breast cancer (Feng et al., 2016), and bladder cancer (Xiong et al., 2017). Intravenous delivery of miR-124 in combination with ENZ sufficiently inhibited prostate tumor growth and increased cell apoptosis (Shi et al., 2015). Mechanistically, miR-124 directly represses *AR* along with *EZH2* and *SRC* through binding to the 3′ UTR regions of these mRNAs (Shi et al., 2015). HDAC inhibitor, OBP-801, induced miR-320a mediated suppression of AR expression through binding to the 3′UTR of *AR* (Sato et al., 2016). Both miR-181c-5p and miR-361-3p could regulate the expression of *AR-V7* but not wild-type *AR* in PCa cells via binding to the specific target sequence in the *AR-V7* 3′UTR (Wu et al., 2019; Liu B. et al., 2020). Besides, exosome transportation of miR-92a-2-5p from macrophages to liver cancer cells could suppress AR expression by directly targeting *AR* 3′UTR and enhance the invasion capacity of liver cancer cells (Liu G. et al., 2020).

### AR Is a Target of lncRNAs

The size of lncRNAs is normally longer than 200 bases, and thus they can fold into complex structures to carry out various functions through interaction with protein, chromatin, and RNA (Schmitt and Chang, 2016; Goodall and Wickramasinghe, 2020). LncRNAs have been reported to regulate gene transcription by recruiting transcription regulators or direct interaction with chromatin, affect protein and mRNA stability through direct binding, and act as sponges for miRNAs (Goodall and Wickramasinghe, 2020). With these diverse regulatory mechanisms, lncRNAs regulate numerous signal pathways and play critical roles in different cellular processes and disease progression. Therefore, lncRNAs have great potentials as biomarkers or therapeutic targets. Currently, many lncRNAs are characterized to participate in PCa progression through direct association with AR protein, DNA, or mRNA (Table 2).

**TABLE 2:** LncRNAs regulating AR.

LncRNAs (References)	Expression in PCa	Molecular mechanisms	Cancer types	Function in cancer cells
*ARLNC1* (Zhang et al., 2018)	↑	Stabilizes the *AR* transcript via RNA-RNA interaction	PCa	Promotes cell growth
*GAS5* (Kino et al., 2010; Pickard et al., 2013; Hudson et al., 2014; Luo et al., 2017)	↓	Interacts with AR protein and suppresses its transcriptional targets	PCa, breast cancer	Promotes cell apoptosis and decreases viability
*HOTAIR* (Zhang et al., 2015)	↑	interacts with AR protein and protects it from degradation	PCa	Promotes cell growth and invasion
*HOXA11-AS-203* (Schmidt et al., 2020)	–	Interacts with AR protein	Melanoma	-
*HOXC-AS1* (Takayama et al., 2020)	↑	Interacts with U2AF2 and promotes *AR* mRNA splicing	PCa	Promotes cell growth
*LBCS* (Gu et al., 2019)	↓	Interacts with hnRNPK and *AR* mRNA to suppress AR translation	PCa	Suppresses cell growth
*MALAT1* (Dai et al., 2019)	↑	Sponges miR-320b and activates AR signaling	PCa	Promotes cell growth, metastasis and invasion
*PCAT1* (Guo et al., 2016)	↑	Interacts with AR protein and regulates its chromosome binding	PCa	Promotes cell growth
*PCGEM1* (Yang et al., 2013; Hung et al., 2014; Prensner et al., 2014c; Zhang et al., 2016)	↑	Interacts with AR protein and enhances its transactivation	PCa	Promotes cell growth
*PlncRNA-1* (Cui et al., 2013; Fang et al., 2016)	↑	Sponges miR-34c and miR-297 and protects *AR* mRNA	PCa	Suppresses apoptosis, promotes cell growth and migration
*PRKAG2-AS1* (Takayama et al., 2020)	↑	Interacts with U2AF2 and promotes *AR* mRNA splicing	PCa	Promotes cell growth
*PRNCR1* (Yang et al., 2013; Prensner et al., 2014c)	↑	Interacts with AR protein and enhances its transactivation	PCa	Promotes cell growth
*SARCC* (Zhai et al., 2016, 2017)	–	Interacts with AR protein and destabilizes it	RCC	Suppresses cell growth
*SLNCR1* (Schmidt et al., 2016, 2020)	–	Interacts with AR protein and regulates its chromosome binding	Melanoma	Promotes melanoma invasion
*SOCS2-AS1* (Misawa et al., 2016)	↑	Interacts with AR protein and regulates its cofactor recruitment	PCa	Promotes cell growth, migration, and suppresses apoptosis
*SRA* (Lanz et al., 1999)	–	Interacts with AR protein and enhances its transactivation	Breast cancer	–
YY1BM (Wu et al., 2020)	–	Blocks the interaction between YY1 and AR protein	Esophageal squamous cell carcinoma	Suppresses cell growth

LncRNA *HOTAIR* was first studied in breast cancer and identified its functionality to reprogram chromatin state by affecting the chromatin occupancy of Polycomb repressive complex 2 (PRC2) and altering histone H3K27 methylation (Wu et al., 2015). Zhang et al. (2015) showed that *HOTAIR* is upregulated in advanced PCa and could reduce AR degradation through directly binding to AR protein which blocked the interaction between AR and MDM2. Interestingly, miR-34a was reported to suppress *HOTAIR* expression through direct binding (Chiyomaru et al., 2013). Coincidentally, AR is a target of miR-34a (Ostling et al., 2011), suggesting *HOTAIR* may also modulate *AR* mRNA expression by acting as a sponge of miR-34a. LncRNA *PCAT1* is a prostate-specific regulator correlated with PCa progression, which was first showed to activate AKT and NF-κB signaling in CRPC through reconfiguring FKPB51-IKKα-PHLPP complex after a direct interaction with FKBP51 (Shang et al., 2019), and suppress BRCA2 expression (Prensner et al., 2014b) and regulate MYC stabilization at the post-transcriptional level (Prensner et al., 2014a). Furthermore, *PCAT1* was reported to interact with AR and LSD1 (Guo et al., 2016). This interaction alters the genomic occupancy of the AR-LSD1 complex, which mainly regulates the transcription of AR target genes through interaction with chromatin (Metzger et al., 2005; Guo et al., 2016). *AR* has also been investigated as an oncogene in human renal cell carcinoma (RCC) (He et al., 2014; Huang Q. et al., 2017), which is consistent with the incidence that RCC is more frequently diagnosed in men than women (Siegel et al., 2021). LncRNA *SARCC* was reported to suppress RCC through binding and destabilizing AR protein and thus concealing AR’s downstream transcriptional targets (Zhai et al., 2016, 2017). *PCGEM1* is a well-known prostate tissue-specific lncRNA associated with high-risk PCa patients (Srikantan et al., 2000; Petrovics et al., 2004; Xue et al., 2013). However, the exact mechanisms of how *PCGEM1* is associated with PCa are conflicting. Yang et al. (2013) reported that *PCGEM1*, together with *PRNCR1* could bind to AR protein and increase its activity through forming an AR-bound enhancer-promoter loop, and both contribute to castration resistance in PCa. However, Prensner et al. (2014c) failed to verify the binding of *PCGEM1* and *PRNCR1* to AR, and they also suggested that neither gene is a component of AR signaling. Then Hung et al. (2014) showed that *PCGEM1* regulates PCa metabolism partially through AR activation, but mainly through promoting chromatin recruitment of c-MYC and activating c-MYC signaling via physical interaction between *PCGEM1* and c-MYC. Interestingly, Zhang et al. (2016) reported that *PCGEM1* could pull down heterogeneous nuclear ribonucleoprotein A1 (hnRNP A1) and splicing factor U2AF65. The *PCGEM1*-hnRNP A1 interaction could suppress hnRNP A1 interaction with *AR* pre-mRNA, while *PCGEM1*-U2AF65 interaction could promote U2AF65 interaction with *AR* pre-mRNA, indicating that *PCGEM1* may participate in the AR signaling by regulation of *AR* mRNA splicing (Zhang et al., 2016). The relationship between *PCGEM1* and AR, and the clinical significance of *PCGEM1* in PCa need further studies.

Growth arrest-specific 5 (*GAS5*) is a lncRNA firstly identified in growth-arrested mammalian cells (Schneider et al., 1988), and overexpression of *GAS5* could induce cell apoptosis and cell cycle arrest in PCa cells (Pickard et al., 2013; Luo et al., 2017; Sun et al., 2017). Intriguingly, *GAS5* was shown to interact with some steroid receptors which share similar response sequences, including glucocorticoid receptor (GR), mineralocorticoid receptor (MR), progesterone receptor (PR), and AR, at their DNA binding domains through *GAS5* contained hairpin RNA glucocorticoid receptor response element (GRE)-mimic and, thereby, inhibit the association of these receptors with their DNA recognition sequence and thus repress the transcriptional activity of these steroid receptors (Kino et al., 2010). These interactions are in a conserved, sequence-specific manner (Hudson et al., 2014). LncRNA steroid receptor RNA activator (*SRA*) could selectively enhance the transactivation of steroid receptors, such as PR, GR, estrogen receptor (ER), and AR, through interaction with their N-terminal, regulatory domain (NTD) (Lanz et al., 1999). Schmidt et al. (2016, 2019) reported another lncRNA, steroid receptor RNA activator-like non-coding RNA (*SLNCR1*), recruits AR to *MMP9* and EGR1-bound genomic loci to regulate melanoma invasion and proliferation. Interestingly, several confirmed AR bound lncRNAs, such as *HOTAIR*, *SRA*, *SLNCR1*, and even *PCGEM1*, all include a conserved region with a similar sequence (*SLNCR1*^609–637^) which is required for AR-lncRNA interaction (Yang et al., 2013; Zhang et al., 2015; Schmidt et al., 2016). Further investigation revealed that AR NTD binds with short, pyrimidine-rich RNA containing at least one CYUYUCCWS motif, and lncRNA *HOXA11-AS-203* which contains such motif was validated to bind with AR NTD (Schmidt et al., 2020). These studies strongly suggested that some lncRNAs containing specific sequences may bind to AR protein and other steroid receptors at the DNA binding domain to compete with the target response elements and suppress their transcriptional activity, or at the N-terminal regulatory domain to modulate their transactivation.

A specific type of lncRNAs called enhancer RNAs (eRNAs) are derived from super-enhancers and have been proven to control mRNA transcription through facilitating enhancer-promoter interaction (Kim et al., 2010; Wang et al., 2011). *KLK3* eRNA (*KLK3e*) is an eRNA produced from the upstream enhancer regions of Kallikrein-related peptidase 3 (*KLK3*) (Hsieh et al., 2014), a well-known AR regulated gene encoding the protein product PSA. *KLK3e*’s expression is induced by AR, and *KLK3e* could scaffold the AR-associated protein complex, the *KLK3* enhancer, and the *KLK2/3* promoter, resulting in enhanced transcriptional activation of nearby *KLK3* and long-distance *KLK2*. daSilva et al. (2018) further identified numerous lncRNAs bound by AR (ARA-lncRNAs), and many of them are also transcriptionally regulated by AR. Further analysis revealed that protein-coding genes adjacent to these ARA-lncRNAs had a significantly greater androgen-induced change in expression than protein-coding genes neighboring lncRNAs not associated with AR, and suppressing the expression of ARA-lncRNA attenuates androgen-induced expression change of protein-coding genes adjacent to the ARA-lncRNA. These ARA-lncRNAs’ transcription start sites (TSSs) are enriched with epigenetic signatures of active enhancers, highlighting hundreds of AR-bound lncRNAs act as cis-regulatory RNA enhancers to control the androgen regulatory program of PCa cells (daSilva et al., 2018). The exact regulation mechanisms and functions of AR-associated eRNAs in PCa cells are still waiting to be fully discovered.

Additionally, several lncRNAs can affect AR signaling through RNA-RNA interaction. LncRNA *ARLNC1* was reported to directly bind to *AR* mRNA 3′ UTR, stabilize *AR* mRNA, and increase the cytoplasmic fraction of *AR* mRNA, thus regulating PCa cell growth and apoptosis (Zhang et al., 2018). LncRNA *LBCS* was also shown to interact with *AR* mRNA and hnRNPK, forming a complex and suppressing AR translation efficiency (Gu et al., 2019). LncRNA metastasis-associated lung adenocarcinoma transcript 1 (*MALAT1*), one of the most studied lncRNAs, was previously reported to bind to EZH2 and enhance EZH2-mediated repression of Polycomb-dependent target genes (Hirata et al., 2015; Wang et al., 2015). *MALAT1* could also function by activating AR signaling through sponging miR-320b which targets *AR* 3′ UTR (Sato et al., 2016; Dai et al., 2019).

Apart from *PCGEM1*, several AR-regulated lncRNAs (*CRPC-lncs*) which are highly expressed in CRPC tissues, were also reported to participate in *AR* mRNA splicing through association with splicing factors (Takayama et al., 2020). Among them, *FAM83H-AS1*, *PRKAG2-AS1*, *HOXC-AS1*, *ELFN1-AS1*, and *ERVK3-1* could interact with U2AF2, which is a component of the U2 complex in spliceosome and regulates *AR* mRNA splicing (Liu et al., 2014; Takayama, 2019; Takayama et al., 2020). Silencing these lncRNAs reduced the nuclear enrichment of U2AF2 and suppressed the association of U2AF2 with *AR* pre-mRNA, resulting in decreased AR expression and inhibited PCa cell growth (Takayama et al., 2020).

A few lncRNAs have been reported to encode short peptides which may play roles as proteins (Anderson et al., 2015; Huang J. Z. et al., 2017). Recently, Wu et al. reported that a Y-linked lncRNA, *LINC00278*, could encode a Yin Yang 1 (YY1)-binding micropeptide, YY1BM. YY1BM could suppress the interaction between YY1 and AR and thus downregulate eEF2K expression and induce apoptosis in human esophageal squamous cell carcinoma (Wu et al., 2020).

### The Regulation of AR by circRNAs

CircRNAs have been recognized as regulatory RNAs (Memczak et al., 2013; Guarnerio et al., 2016), and exhibit critical roles through mechanisms like lncRNAs. CircRNA could inhibit miRNA target degradation as sponges (Hansen et al., 2013; Zheng et al., 2016), bind to proteins, RNAs, and DNAs to affect gene transcription (Li et al., 2015; Yang et al., 2017), RNA splicing (Conn et al., 2017), and translation (Li et al., 2020), and serve as protein scaffold containing different binding sites (Du et al., 2017). CircRNA also has its specific regulatory mechanism distinct from lncRNA. CircRNAs’ unique circularization structure lacking open ribonucleotide end may resist the RNA cleavage by miRNA recruited exonuclease, thus stabilizing miRNAs after binding (Piwecka et al., 2017; Chen S. et al., 2019). CircRNA profiling through ribosomal-depleted RNA sequencing has identified many circRNAs that are differentially expressed between normal and cancerous prostate tissues (Zheng et al., 2016; Chen S. et al., 2019). Among them, circRNA-17 is lower expressed in higher grade PCa tissues, and suppressing circRNA-17 could increase the expression of AR-V7, and enhance the resistance to anti-AR therapy. Further investigation revealed that circRNA-17 could bind and stabilize miR-181c-5p, which targets the 3′UTR of AR-V7 (Wu et al., 2019). Since circRNA is a relatively new research field of ncRNAs, more investigations about circRNAs are needed to explore and elucidate their exact roles in tumorigenesis.

## ncRNAs as AR Targets

As a critical hormonal transcription factor, AR can exhibit its function through direct binding to ARE located at enhancers and promoters of its targets. Genomic occupation of AR and profiles of androgen-responsive genes have been defined through Chromatin Immunoprecipitation (ChIP)-on-chip, ChIP-seq assays (Jin et al., 2013). AR directly targeted miRNAs (Takayama et al., 2011; Pasqualini et al., 2015) and lncRNAs (Zhang et al., 2018; Takayama et al., 2020) in PCa cells have also been systematically identified by combined analysis of androgen dysregulated miRNA and lncRNA expression data from microarray or RNA sequencing with AR genome-wide binding information (Table 3).

**TABLE 3:** AR regulated ncRNAs.

ncRNAs (References)	Regulation by AR	Molecular mechanisms	Cancer types	Function in cancer cells
**miRNAs**		**Targeted genes**		
Let-7d (Ramberg et al., 2011)	↑	*PBX3*	PCa	–
miR-1 (Liu Y. N. et al., 2015; Chen W. Y. et al., 2017)	↑	*ZBTB46*, *SRC*	PCa	Inhibits metastasis
miR-101 and miR-26a (Cao et al., 2010)	↑	*EZH2*	PCa	Inhibits proliferation and invasiveness
miR-125b (Rajabi et al., 2011)	↑	*MUC1*, *BAK1*	PCa	Promotes cell growth
miR-135a (Kroiss et al., 2015)	↑	*ROCK1*, *ROCK2*	PCa	Inhibits invasiveness
miR-141 (Waltering et al., 2011)	↑	-	PCa	Promotes cell growth
miR-148a (Fujita et al., 2010; Murata et al., 2010)	↑	*CAND1* (Murata et al., 2010), *MSK1* (Fujita et al., 2010)	PCa	Promotes LNCaP cell growth (Murata et al., 2010), Suppresses PC3 cell growth, and invasiveness (Fujita et al., 2010)
miR-185-5p (Huang Q. et al., 2017; Zhu et al., 2019)	↑	*CSF-1*, *VEGFC*, *HIF2α*	RCC	Increases RCC cell metastases to lung and liver, while suppresses the lymph nodes metastases
miR-193a-3p (Jia et al., 2017)	↑	*AJUBA*	PCa	Promotes cell migration
miR-21 (Ribas et al., 2009; Mishra et al., 2014)	↑	*TGFBR2*	PCa	Promotes cell growth
miR-216a (Chen et al., 2012)	↑	*TSLC1*	HCC	Promotes cell growth and migration
miR-22, miR-29a, and miR-17-92 cluster (Pasqualini et al., 2015)	↑	*LAMC1*, *MCL1*	PCa	Deceases cell viability and migration
miR-29 (Takayama et al., 2015)	↑	*TET2*	PCa	Promotes cell growth and migration
miR-31 (Lin et al., 2013)	↓	*AR*	PCa	Suppresses cell growth
miR-32 and miR-148a (Jalava et al., 2012)	↑	*BTG2*, *PIK3IP1*	PCa	Reduces apoptosis or promotes cell growth
miR-99a/let7c/125b-2 cluster (Sun et al., 2014)	↓	*IGF1R*	PCa	Suppresses cell growth
miR-421 (Ostling et al., 2011; Meng et al., 2016)	↓	*AR* (Ostling et al., 2011), *NRAS*, *PRAME*, *CUL4B*, and *PFKFB2* (Meng et al., 2016)	PCa	Suppresses cell growth, induces cell cycle arrest, reduces glycolysis, and inhibits migration
**lncRNAs**				
*ARNILA* (Yang et al., 2018)	↓	Sponges miR-204 to facilitate Sox4 expression	Breast cancer	Promotes migration, invasion and EMT
AR-Associated lincRNAs (daSilva et al., 2018)	↑	Scaffolds AR-dependent looping complex	PCa	–
*CTBP1-AS* (Takayama et al., 2013)	↑	Represses CTBP1 by recruiting PSF together with histone deacetylases	PCa	Promotes cell growth
*DANCR* (Jia et al., 2016)	↓	Represses TIMP2/3 expression by mediating the binding of EZH2 on their promoters	PCa	Promotes cell invasion and metastasis
*DRAIC* (Sakurai et al., 2015)	↓	-	PCa	Suppresses cell migration and invasion
*KLK3e* (Hsieh et al., 2014)	↑	Scaffolds AR-dependent looping complex	PCa	Promotes cell growth
*Linc00304* (Zhang et al., 2019)	↓	Promotes CCNA1 expression	PCa	Promote cell growth and cell cycle progression
*Linc00844* (Lingadahalli et al., 2018)	↑	Indirectly modulates AR binding to chromatin	PCa	Suppresses cell migration and invasion
*Linc01503* (He et al., 2020)	↑	Recruits SFPQ and activates FOSL1	Nasopharyngeal carcinoma (NPC)	Promotes cell growth, migration, and invasion
*LncRNA-p21* (Luo et al., 2019b)	↓	Interacts with EZH2 and enhances STAT3 methylation	PCa	Promotes ENZ induced neuroendocrine differentiation (NED)
*PCAT18* (Crea et al., 2014)	↑	–	PCa	Promotes cell growth, migration, and invasion
*PCAT29* (Malik et al., 2014; Sakurai et al., 2015)	↓	–	PCa	Suppresses cell migration and invasion
*POTEF-AS1* (Misawa et al., 2017)	↑	Represses Toll-like receptor signaling	PCa	Promotes cell growth and suppresses apoptosis
*SOCS2-AS1* (Misawa et al., 2016)	↑	Interacts with AR protein and regulates its cofactor recruitment	PCa	Promotes cell growth and suppresses apoptosis
*TMPO-AS1* (Huang et al., 2018)	↓	–	PCa	Promotes cell growth and migration
**circRNAs**				
circAR3 (Luo et al., 2019a)	↓	Encoded by *AR* gene	PCa	No effect on cell growth and invasion
AR-circRNAs (Cao et al., 2019)	↓	Encoded by *AR* gene	PCa	–
circHIAT1 (Wang k. et al., 2017)	↓	Stabilizes miR-195-5p/29a-3p/29c-3p	ccRCC	Suppresses cell migration and invasion
circRNA7 (Bao S. et al., 2020)	↓	Sponges miR-7-5p and increases VE-cadherin and Notch4	HCC	Promotes vasculogenic mimicry formation
circZMIZ1 (Jiang et al., 2020)	↑	Increases expression of AR and AR-V7	PCa	Promote cell growth and cell cycle progression

Takayama et al. (2011) integrated 5′-cap analysis of gene expression (CAGE) and ChIP-on-chip analysis and identified a cluster of androgen-inducible miRNAs in LNCaP cells, including miR-100, miR-125b, miR-21, miR-218-1, miR-218-2, miR-221, miR-222, and let-7c, which are all located adjacent to androgen receptor binding sites (Takayama et al., 2011). Among them, miR-21 has been verified as one of 16 AR-responsive miRNAs (Ribas et al., 2009). Interestingly, miR-21 may indirectly increase AR expression by decreasing PTEN, forming a positive regulation loop between AR and miR-21 (Mishra et al., 2014). MiR-125b was shown to be induced by AR and partially involved in AR’s downregulation of MUC1 by targeting *MUC1* 3′ UTR (Rajabi et al., 2011). Sun et al. (2014) reported that miR-125b-2, let-7c, and miR-99a are a cluster of miRNAs from the same host gene, and were all repressed following androgen activation in LNcaP cells, which is in contrast to Takayama et al.’s result probably due to different conditions of androgen treatment and tissue culture. Pasqualini et al. (2015) performed AR ChIP-seq and miRNA host gene array analysis after AR stimulation in DUCaP cells, and successfully identified 32 miRNA host genes that were significantly regulated and bound by AR. MiR-22 and miR-29a are significantly increased by AR activation in a time-dependent manner, and both of them are higher expressed in benign prostate tissues when compared to tumor tissues. MiR-125b, miR-22, and miR-29a/b were later examined to mediate AR’s repression of TET2 in PCa cells (Takayama et al., 2015). Besides, AR-induced miR-26a together with miR-101 both target *EZH2* at the 3′ UTR region and thus are involved in AR’s regulation of EZH2 (Cao et al., 2010). Murata et al. (2010) identified androgen-responsive miRNAs in LNCaP cells through short RNA sequencing and the expression of miR-148a, miR-141, and miR-200a along with miR-125b, miR-22, and miR-29b were all increased after R1881 treatment in LNCaP cells (Murata et al., 2010). The function of miR-148a is complex in prostate cancer progression. MiR-148a was highly expressed in PCa patients and significantly correlated with biochemical recurrence of PCa independent of PSA values (Al-Qatati et al., 2017), and it was shown to promote LNCaP cell growth through targeting *CAND1* 3′ UTR (Murata et al., 2010). However, overexpression of miR-148a precursor suppressed androgen-refractory PC3 cell growth (Fujita et al., 2010). Another report also suggested miR-148a exhibited tumor suppressor roles in several common cancers (Lujambio et al., 2008). As more AR-regulated miRNAs in PCa and other types of cancers were reported, it is clear that AR’s function is partially mediated by AR-induced oncogenic miRNAs and -inhibited tumor-suppressive miRNAs (Table 3).

Similarly, many AR-regulated lncRNAs have also been identified in PCa cells (Table 3). Misawa et al. (2016) identified 5 lncRNAs induced by androgen through RNA sequencing. Surprisingly, one of these 5 lncRNAs, *SOCS2-AS1*, was shown to interact with AR protein and modulate AR activity by regulating cofactor recruitment, leading to a positive regulation loop in PCa cells. Zhang et al. (2018) performed an integrative transcriptomic analysis in PCa tissues combined with AR ChIP-seq, resulting in the identification of AR-regulated clinically relevant lncRNAs and *ARLNC1* was identified as one of the AR-regulated lncRNAs that regulates *AR* mRNA stability (Zhang et al., 2018). Another lncRNA in the list is *PRCAT38*, which was later proven to share enhancers with *TMPRSS2*, and both of them are activated by AR/FOXA1 binding (Chen Z. et al., 2019). *PCAT29* is a PCa-associated lncRNA suppressed by DHT and knocking down *PCAT29* increases PCa cell proliferation and migration (Malik et al., 2014). Takayama et al. (2020) identified AR-regulated lncRNAs which are highly expressed in CRPC tissues. Among the list, *PRKAG2-AS1* was suppressed by AR activation, while *HOXC-AS1* was induced by AR activation, and both lncRNAs play essential roles in *AR* mRNA splicing through interaction with *AR* splicing factor, U2AF2.

Several circRNAs directly regulated by AR have also been reported (Table 3). CircRNA-ZMIZ1 is upregulated in PCa patients’ plasma samples than in corresponding normal samples (Jiang et al., 2020). CircRNA-ZMIZ1 expression is increased by androgen activation, and silencing circRNA-ZMIZ1 induces PCa cell growth inhibition and cell cycle arrest. ChIP-seq and luciferase assay confirmed that AR suppresses the expression of circRNA-HIAT1 in clear cell renal cell carcinoma (ccRCC). CircRNA-HIAT1 serves as a “reservoir” to stabilize miR-195-5p/29a-3p/29c-3p that target *CDC42*, thus indicating AR promotes ccRCC through regulating circHIAT1/miR-195-5p/29a-3p/29c-3p/*CDC42* axis (Wang k. et al., 2017). Interestingly, *AR* can be transcribed into several circRNAs due to alternative RNA splicing (Cao et al., 2019; Luo et al., 2019a). The expression of these *AR*-transcribed circRNAs are positively correlated with linear *AR* transcripts and can be detected in plasma samples from metastatic castration-resistant PCa (mCRPC) patients and may serve as biomarkers of high-risk primary PCa (Cao et al., 2019; Luo et al., 2019a).

## Discussion

### Crosstalk Between ncRNAs in AR Regulatory Network

**FIGURE 1:**
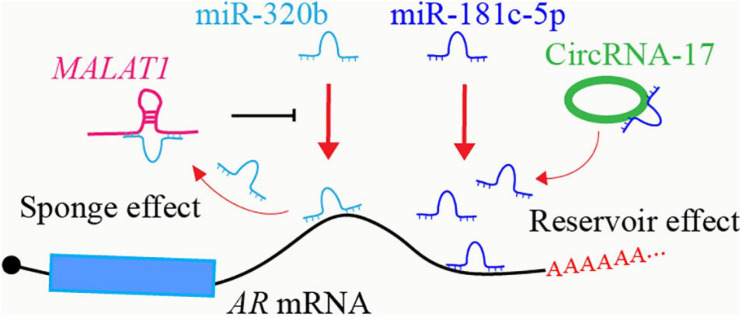
Crosstalk between ncRNAs in AR regulatory network. LncRNA *MALAT1* acts as a sponge to inhibit miR-320b-targeted *AR* mRNA degradation. CircRNA-17 binds to and stabilizes miR-181c-5p, enhancing miR-181c-5p-targeted *AR* mRNA degradation.

Some lncRNAs and circRNAs share similar RNA sequences as the miRNA targeted mRNA, and then they could act as miRNA sponges to diminish miRNA-induced mRNA degradation (Hansen et al., 2013; Goodall and Wickramasinghe, 2020). For example, lncRNA *MALAT1* could decrease miR-320b mediated *AR* mRNA degradation through competitively binding to miR-320b (Dai et al., 2019; Figure 1). Some circRNA-miRNA interactions may form RNA duplex resistant to RNA cleavage, thus stabilizing miRNAs (Piwecka et al., 2017; Chen S. et al., 2019; Wu et al., 2019). CircRNA-17 suppresses the expression of AR-V7 by binding and stabilizing miR-181c-5p which induces the degradation of *AR-V7* through targeting its 3′ UTR region (Wu et al., 2019; Figure 1). Both lncRNAs and circRNAs can also serve as scaffolds to mediate interactions between proteins and RNAs. LncRNAs and circRNAs might also mutually affect the binding with the same targets, which may be due to the RNA sequence similarity between lncRNAs and circRNAs, or RNA structure-induced protein conformational change.

### Feedbacks Between AR and ncRNAs

**FIGURE 2:**
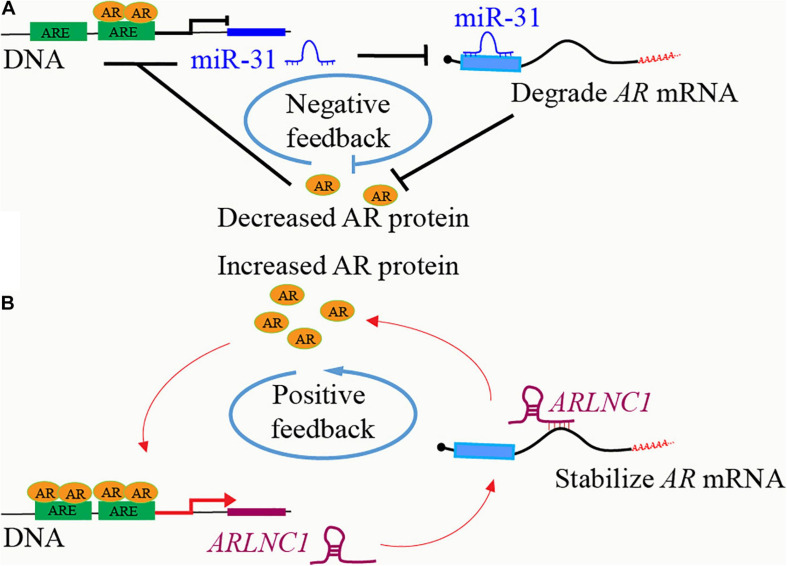
Feedbacks between AR and ncRNAs. **(A)** AR binds to the promoter region of lncRNA *ARLNC1* and induces its expression. *ARLNC1* binds to and stabilizes *AR* mRNA, which increases AR expression and further activates the transcription of itself, *ARLNC1*, forming a positive feedback regulation loop. **(B)** AR binds to the promoter region of miR-31 and suppresses its expression. MiR-31 induces *AR* mRNA degradation via targeting the coding region of *AR* mRNA, thus forming a negative feedback regulation loop.

As one of the most important regulators in PCa, the expression of AR is precisely controlled by various factors through different mechanisms in different stages, including feedback regulation loops between AR and ncRNAs. Many ncRNAs regulate AR expression at transcription and post-transcription levels, while they are also regulated by AR. MiR-31 inhibits AR expression by directly targeting the *AR* mRNA coding region, while miR-31 itself is suppressed as an AR repressive target, thus forming a negative feedback loop to promote PCa (Lin et al., 2013; Figure 2A). AR increases the expression of miR-21, which in turn increase AR expression and activity probably via the down-regulation of PTEN (Ribas et al., 2009; Mishra et al., 2014). Both AR-induced *HOXC-AS1* and AR-repressed *PRKAG2-AS1* can regulate *AR* mRNA splicing and promote AR expression (Takayama et al., 2020). The expression of *ARLNC1* is increased after AR binding to the *ARLNC1* promoter region. *ARLNC1* further stabilizes *AR* mRNA and promotes AR expression through binding to *AR* mRNA 3′ UTR, thus forming a positive regulation loop in PCa cells (Zhang et al., 2018; Figure 2B).

### Steroid Receptors and lncRNAs

**FIGURE 3:**
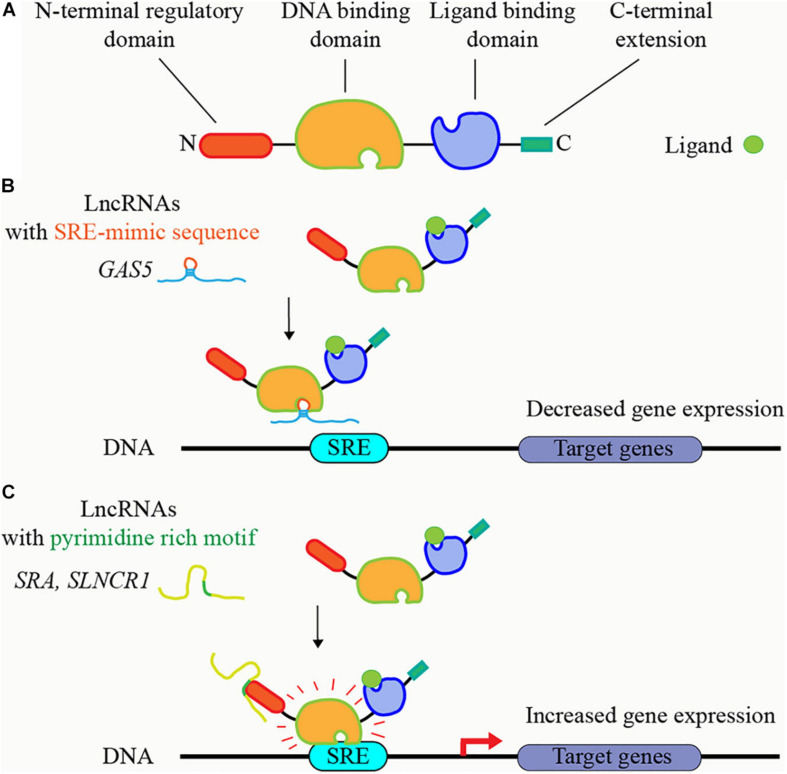
LncRNAs bind to steroid receptors and regulate their activities. **(A)** Principal functional domains of steroid receptors. The steroid receptors have N-terminal regulatory domain, central DNA binding domain, ligand-binding domain, and C-terminal extension. **(B)** LncRNAs containing steroid receptor responsive element (SRE) mimic RNA sequence, such as *GAS5*, block the binding of steroid receptors to SRE DNA sequence, and suppress steroid receptors’ transcriptional activity. **(C)** LncRNAs containing short, pyrimidine-rich RNA motif, such as *SRA* and *SLNCR1*, bind to steroid receptors at N-terminal, regulatory domain, and increase steroid receptors’ transcriptional activity.

Several lncRNAs have been shown to regulate steroid receptors, including GR, MR, PR, AR, and other nuclear receptors, through directly binding in a conserved sequence-specific manner (Kino et al., 2010; Hudson et al., 2014; Liu et al., 2016). These lncRNAs bind to these receptors at two protein domains: DNA binding domain, and the N-terminal, regulatory domain (Figure 3A). *GAS5* contained a GRE-mimic hairpin RNA sequence and thus can bind to GR DNA domain, block GR’s binding to GRE DNAs, and suppress GR-induced gene transcription (Kino et al., 2010; Hudson et al., 2014; Schmidt et al., 2016; Figure 3B); Figure 3B). On the other hand, several AR-bound lncRNAs share short, pyrimidine-rich RNA motif (CYUYUCCWS) that are required for the interaction with steroid receptors at N-terminal, regulatory domain, such as *HOTAIR*, *SRA*, *SLNCR1*, *HOXA11-AS-203*, and *PCGEM1* (Lanz et al., 1999; Yang et al., 2013; Zhang et al., 2015; Schmidt et al., 2016; Figure 3C); Figure 3C). The discovery of ncRNAs including circRNAs containing steroid receptor responsive element (SRE) mimic RNA sequences, or the pyrimidine-rich RNA motif (CYUYUCCWS) is of great interest to identify new mechanisms in various diseases that ncRNAs regulate steroid receptors through binding to steroid receptors. These regulation mechanisms may also apply to the ncRNAs that bind to other transcription factors.

### Clinical Implications of ncRNAs in PCa

Increasing research of ncRNAs has greatly revolutionized our understanding of RNA biology. More and more evidence showed that ncRNAs have critical functions in diverse diseases. MiRNA, lncRNA, and circRNA are ubiquitously expressed throughout the body, and they can readily be measured from various human samples, including serum, saliva, and urine (Weber et al., 2010; Iyer et al., 2015; Vo et al., 2019). Many ncRNAs play important roles in PCa and their expressions are correlated with different clinicopathological characteristics of PCa patients. These ncRNAs hold great promises as biomarkers and therapeutic targets in clinical applications. Several single miRNA and panels of miRNAs combinations from plasma or tissue samples of PCa patients have shown more extraordinary diagnostic performance than PSA (Kachakova et al., 2015; Kelly et al., 2015). At the same time, serum miR-210 level is notably correlated with the change in PSA level during treatment among metastatic CRPC patients (Cheng et al., 2013). MRX34, a synthetic miRNA mimic of miR-34a that directly regulates at least 24 known oncogenes including AR, is the first miRNA mimic in clinic application (Bouchie, 2013). In phase 1 clinical trial (NCT01829971), MRX34 was delivered in patients with advanced solid tumors by a liposome technology named Smarticles, which demonstrated exciting proof-of-concept for miRNA-based cancer treatment but unfortunately failed due to serious adverse events (Hong et al., 2020). On the other hand, lncRNA *PCA3* is specifically overexpressed in most PCa cancer patients (Bussemakers et al., 1999) and has been approved by the FDA as a PCa diagnostic marker in the urine of PCa patients (de Kok et al., 2002; Deras et al., 2008), but its use for assessing response to ADT in advanced PCa is limited (Martinez-Pineiro et al., 2014). Some other lncRNAs have been identified as biomarkers for metastatic PCa, such as *PCAT18* (Crea et al., 2014) and *SChLAP1* (Prensner et al., 2014d). Candidate circRNAs were also identified and detected in urine to serve as biomarkers for PCa (Vo et al., 2019).

Antisense oligonucleotides (ASOs) as an RNA-based therapeutic approach can induce gene silencing through RNase H-mediated degradation of target RNAs. It has shown improved target specificity and stability as well as tolerated toxicity after significant advancements in the design, chemical modifications, and delivery (Verma, 2018). Several ASO-based drugs have been approved by the FDA for the treatment of different human diseases (Dhuri et al., 2020). For PCa treatment, ASOs targeting *Bcl-2* mRNA (Oblimersen- G3139) (NCT00085228) and *Clusterin* mRNA (Custirsen- OGX011) (NCT01188187) had been evaluated in PCa human patients in Phase II and III clinical trials, but both failed due to major toxic events or no significant survival improvement (Sternberg et al., 2009; Beer et al., 2017). Additionally, ASOs targeting *Hsp27* mRNA (Apatorsen- OGX-427) (NCT01120470) and *AR* mRNA (ARRx- AZD5312) (NCT03300505) are currently under Phase I clinical trials. In addition to targeting the protein-coding mRNAs, ASO targeting lncRNA *MALAT1* dramatically prevented lung cancer metastasis in a pulmonary metastatic mouse model (Gutschner et al., 2013), showing attractive potentials for developing ASO drugs targeting functional ncRNAs to treat PCa. More preclinical investigations for ASO targeting ncRNAs are needed to enable ASO-based prostate cancer treatment in the near future.

### LncRNA Studies Through *in vivo* Mouse Models

Most lncRNAs’ functions and mechanisms were revealed through knocking down strategies from *in vitro* study until to date, and several lncRNAs are proven to be necessary for life and brain development through the studies in the knockout mouse model (Sauvageau et al., 2013; Nakagawa et al., 2014). On the other hand, accumulating evidence showed that inactivating the same lncRNAs in mouse models resulted in no phenotype, and even opposite effects for some lncRNAs (Bassett et al., 2014; Sun and Ma, 2019). Bassett et al. (2014) summarized the results of *in vivo* studies of 30 lncRNAs through different inactivation strategies from 17 groups. Among them, lncRNA *MALAT1* was inactivated through 3 different strategies: deleting 3kb genomic region covering the 5′ end of *MALAT1* and its promoter (Zhang et al., 2012), removing the entire 7kb *MALAT1* gene (Eissmann et al., 2012), and premature transcriptional termination by inserting *lacZ* and polyadenylation sequences downstream of the transcriptional start site of *MALAT1* (Nakagawa et al., 2012). All these *MALAT1*-deficient mice from these 3 studies were viable and fertile without significant changes in mice development and growth, and global gene expression, which argues against the in vitro and xenograft studies that demonstrated *MALAT1*’s role in promoting cell proliferation and metastases through regulating pre-mRNA splicing (Tripathi et al., 2010), coordinating gene transcription (Yang et al., 2011; Wang et al., 2015), and acting as competitive endogenous RNA (Wang y. et al., 2017; Dai et al., 2019). Recently, Kim et al. (2018) investigated *MALAT1*’s role in a transgenic mouse model of breast cancer in which the *MALAT1* gene was inactivated through premature transcriptional termination by inserting lacZ and polyadenylation sequences as Nakagawa et al. (2012). Targeted inactivation of *MALAT1* in this breast cancer mouse model doesn’t affect breast tumor growth, but surprisingly promotes breast cancer lung metastasis (Kim et al., 2018), which is consistent with some other reports that suggest *MALAT1* functions as a tumor suppressor (Xu et al., 2015; Cao et al., 2016; Han et al., 2016; Latorre et al., 2016; Kwok et al., 2018). Importantly, the metastatic-promoting effect of *MALAT1* insertional inactivation can be reversed by genetical re-expression of *MALAT1*, and targeted transgenic overexpression of *MALAT1* in mice inhibits breast cancer metastasis (Kim et al., 2018), strongly suggesting that the lncRNA *MALAT1* suppresses breast cancer metastasis.

There are several possible reasons to explain why some *in vivo* mouse models failed to validate lncRNAs’ function discovered from *in vitro* and xenograft studies. First, most lncRNAs were studied in cells through the knocking down methods mediated by short-hairpin RNA (shRNA) or siRNA without appropriate rescue assays. Silencing nuclear lncRNAs requires the nuclear enrichment of AGO2 and RNA interference (RNAi) factors Dicer, TRBP, and TRNC6A/GW182 (Gagnon et al., 2014), while AGO2’s nuclear distribution depends on cell type and tissue context (Sharma et al., 2016), and thus results from knocking down lncRNAs in cells lacking nuclear distribution of AGO2 are questionable. Secondly, several lncRNAs were silenced in cells and mouse through ASO treatment. However, the delivery of ASO to the targeted cells and organs is still a challenge, and it is not clear whether ASO could efficiently degrade the nascent RNAs. Besides, the potential off-target effects of ASO may lead to non-specific results (Deleavey and Damha, 2012). Thirdly, loss of function approaches in cells and mouse models through large size gene deletion or clustered regularly interspaced short palindromic repeats (Crispr)-Cas9 knocking out may also delete the neighboring genes and destroy the regulatory elements for other genes. Some lncRNAs function through *cis* mechanisms to regulate their neighboring genes. The effect of destructing these regulatory elements located in the lncRNA genome loci and neighboring genes prevails against the effect of lncRNA loss (Yin et al., 2015). LncRNA inactivation induced by transcriptional terminator insertion abrogates lncRNA transcription with minimal disruption of genomic sequences and mice phenotypes induced by insertional inactivation of lncRNAs can be rescued by re-expression of lncRNAs (Bond et al., 2009; Berghoff et al., 2013; Grote et al., 2013). Fourthly, even though lncRNA is inactivated through the same method, different mouse models may display different phenotypes. Insertional inactivation of *MALAT1* in MMTV-PyMT mouse which is a transgenic model of metastatic breast cancer, induced significantly increased lung metastasis of breast cancer cells (Kim et al., 2018), while insertional inactivation of *MALAT1* in mouse with normal physiological condition showed no apparent phenotype (Nakagawa et al., 2012), suggesting *MALAT1* is dispensable for development but plays important roles in suppressing breast cancer metastasis. Taken together, it is critical to choose the proper method to generate lncRNA depleting cells and mouse model based on lncRNA’s cellular distribution, genome localization and its function mechanism, and it is also important to take rescue experiments into consideration when investigating lncRNAs’ function through loss of function methods (Bassett et al., 2014; Kopp and Mendell, 2018).

## Conclusion

A growing number of novel discovered ncRNAs and various research has revealed the crucial roles of ncRNAs in different disease processes. Many ncRNAs have been verified to participate in PCa initiation or progression by regulating or mediating AR signaling. These ncRNAs hold great potentials as diagnostic biomarkers and therapeutic targets. Given that ncRNAs consist of the majority of the human transcriptome, a long journey in the understanding of ncRNAs, especially lncRNAs, circRNAs, and other ncRNAs, is yet to be achieved. Further investigations based on high-throughput sequencing technology and integrative bioinformatics analysis will enable the discovering of new functional ncRNAs and their regulation mechanisms, and these works will further promote the development of effective therapeutic strategies for PCa.

## Author Contributions

YY and QC developed the concept of the review. YY and KL collected the related publications and drafted this manuscript. QL and QC helped to revise this manuscript. All authors read and approved the final manuscript.

## Conflict of Interest

The authors declare that the research was conducted in the absence of any commercial or financial relationships that could be construed as a potential conflict of interest.
